# Social perception inferences of computer-generated faces: an Asian Indian and United States cultural comparison

**DOI:** 10.3389/fpsyg.2023.1174662

**Published:** 2023-07-24

**Authors:** Anthony Stahelski, Mary Katherine Radeke, Maxie Reavis

**Affiliations:** ^1^Department of Psychology, Central Washington University, Ellensburg, WA, United States; ^2^College of Osteopathic Medicine, Pacific Northwest University of Health Sciences, Yakima, WA, United States

**Keywords:** facial expressions, social perception inferences, cross-cultural facial expressions, cross-cultural, facial structure

## Abstract

Results from research with computer-generated faces have demonstrated that participants are able to make different trait inferences to different generated faces. However, only a few studies using computer-generated faces with cross-cultural samples have been done. This study compared the facial trait inference results from India and the United States, using three validated neutral expression computer-generated faces from the University of Chicago Perception and Judgment Lab database as facial stimuli. The three faces varied in perceived threat. Participants were asked about the attractiveness, pleasing-ness (to look at), honesty, and potential threat in each of the three faces. Results indicated that participants from both cultural samples made the same inferences to the three faces; participants rated the attractiveness, pleasing-ness, and honesty highest in the low threat face and lowest in the high threat face. Indian participants perceive the high threat face to be less threatening than the United States participants. Participants were also asked about the emotional expression on each of the faces, even though the faces were presumably neutral. United States participants were significantly more likely to indicate that the faces in all three threat conditions were emotionally neutral, compared to Indian participants, reflecting a cultural In-group bias, in which members of a culture are more accurately able to identify expressions on faces from their own culture.

## 1. Introduction

The idea that people derive insights about others from their faces was first formally investigated by Charles Darwin. Darwin hypothesized that human facial expressions evolved from animal responses that these expressions connote emotions, and that human facial expression-emotion connections are human universals (Darwin, 1872/1998). Darwin’s hypotheses were unexplored with modern research methodology until the 1960s, when Paul Ekman and Wallace Friesen began systematically studying the possible universality of emotional facial expressions (Ekman and Friesen, 1969). Their research supports Darwin’s universality hypothesis for six facial expressions and their corresponding emotion labels: anger, disgust, happiness, fear, sadness, and surprise (Ekman and Frisen, 1971). Further 20th Century, research supported the cross-cultural recognition of the six facial expressions (Izard, 1971; Ekman and Frisen, 1986; Ekman and Heider, 1988; Matsumoto, 1992). Scherer et al. (1988) examined reactions to the six emotional facial expressions across 27 countries, and significant cross-cultural participant agreement regarding the six expression and emotion connections was observed. More recently, Elfenbein and Ambady (2002a) confirmed the cross-cultural agreement regarding the six facial expression-emotion connections.

A study comparing Indian and United States participants (Hejmadi et al., 2000) used videotaped dance sequences expressing 10 different classic Hindu emotions (anger, disgust, fear, heroism, humor-amusement, love, peace, sadness, shame-embarrassment, and wonder) as stimuli for American and Indian participants. The dance sequences were all performed by a Hindu Indian dancer, showing her face, hands and body movements. Participants were assigned randomly to either a fixed or free-response format for identifying the emotion depicted in the videotaped sequence. Results indicated that both Indian and United States participants had high levels of accurate emotional identification, with no significant differences between the two groups, indicating support for the universality hypothesis.

Research on the universality hypothesis has been extended to perceptions and traits other than emotions for an obvious reason: people use faces to assess the social characteristics and personality traits of others. Researchers have examined facial inferences for a number of characteristics and traits, and they have concluded that there is cross-cultural agreement about which inferences go with which faces. For example, individuals with smiling faces are cross-culturally perceived to be more attractive and trustworthy than those who are not smiling (Cunningham et al., 1995; Zebrowitz et al., 2003; Zebrowitz and Montepare, 2008; Xu et al., 2012).

However, even though the facial recognition of emotions and other traits has been shown in these studies to be apparently universal, the universalist position has not gone unchallenged (Russell, 1994; Gendron et al., 2014). Results from numerous cross-cultural inferencing studies, including the ones that basically support the universalist hypothesis, have shown that a varying number of participants do not always infer the same emotions and traits from the same faces (Walker et al., 2011; Gendron et al., 2014). Culture impacts the facial inferencing process in two ways. First, there is evidence that members of a culture can more accurately identify emotions and traits from the faces of members of their own culture; this is known as the In-group advantage (Elfenbein and Ambady, 2002b). Second, cultures vary on a number of dimensions (Hofstede, 2001), and the most researched dimension is collectivism vs. individualism. Both of these cultural influences are relevant to our study, which compares the facial inferences made by Indian and United States participants.

A recent study (Mishra et al., 2018) compared Indian and Dutch participant responses to various emotional expressions on static Dutch faces. Results indicated that the accuracy of expression identification was high and similar for both participant groups. However, there was a significant In-group accuracy advantage for Dutch participants looking at Dutch faces. In addition Indian participants particularly misperceived the negative expressions of anger and fear as something less negative. The authors explain this finding by hypothesizing that collectivist cultures like India discourage expressing negative emotions in order to preserve group cohesion and harmony.

The present study similarly compares Indian and Western (United States) samples, using computer-generated faces as stimuli instead of real human faces taken from facial databases (Oosterhof and Todorov, 2008; Todorov et al., 2015). The purpose of this study is to further understand the contributions of evolution and culture to the facial inference process by assessing the emotional and social trait inferences made by participants in two cultural samples to computer generated faces. The human face and head contain numerous features, which can vary between individuals, making systemic comparisons of variables difficult. Todorov and colleagues have created computer-generated faces eliminating various features (such as hair) as a solution to this problem. The faces were created to be emotionally expressionless (neutral) and ambiguous as to age and gender. Additionally, Todorov and colleagues created all the faces to resemble Caucasian/European faces to eliminate race as a variable (Oosterhof and Todorov, 2008). These steps were taken to assess the contributions of specified facial structure features, such as skin reflectance, eyebrow placement, eye shape, and lip thickness, to the facial inference process. Research using these expressionless presumably neutral faces has demonstrated that participants use facial structure features to make both emotion and social trait inferences (Todorov and Oh, 2021). One of the inferences studied has been perceived threat. Different computer-generated expressionless faces varying in structural features are perceived by United States participants to vary in threat (Olivola et al., 2014). Will the two cultural groups utilized in this study perceive the same (or different) threat variations in these faces?

### 1.1. Hypotheses

Hypothesis 1: the Indian and US participants will perceive the same social perception differences (*attractiveness*, *honesty*, *pleasing to look at*, and *threat*) between the low, neutral, and high threat computer-generated faces, indicating that expressionless facial structure features are used cross-culturally to make the same facial inferences.
Hypothesis 2: since the neutral faces were created to look like individuals of European descent, United States participants will have an In-group advantage for accurately perceiving emotional neutrality in the faces: Indian participants will perceive more emotionality and less neutrality in all three faces compared to the United States participants.

## 2. Methods

### 2.1. Participants

Two hundred India participants (73% male, average age range 26–35 years); and 200 United States participants (50% male, average age range 31–40 years). Participants responded to a demographic question by selecting their age range rather than entering their actual age. See Table 1 for percent of age range for India and United States participants. All participants were recruited using Amazon’s Mechanical Turk (MTurk) recruitment service (Bosch, 2015) and were compensated for participation.

**Table 1:** Percent of age range for India and United States participants.

	18–20 years	21–25 years	26–30 years	31–35 years	36–40 years	41–45 years	46–50 years	51+ years	No answer
India	1.5%	18%	38%	21%	9%	5%	4%	3%	0.5%
United States	1%	12%	25%	22%	11%	8.5%	8%	12.5%	0%

### 2.2. Images

**Figure 1:**
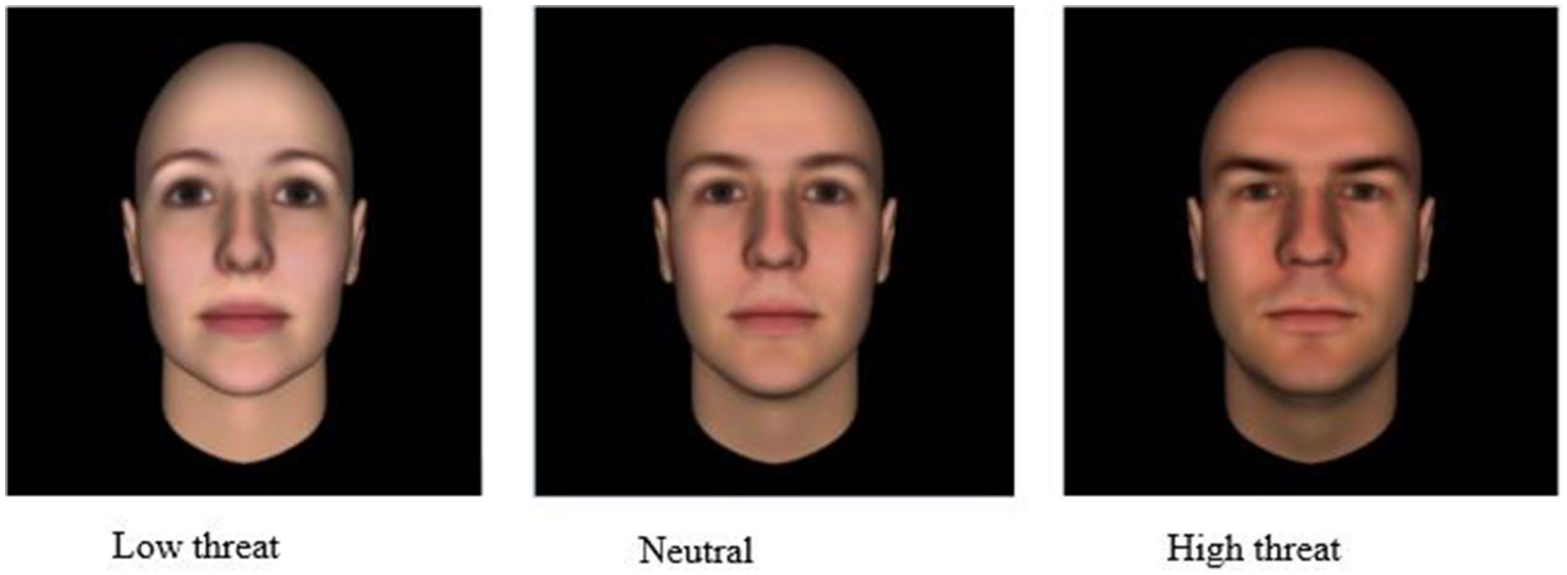
Computer generated threat faces; low threat, neutral, and high threat (reproduced with permission from Dr. Alexander Todorov: https://tlab.uchicago.edu/databases/).

The three facial images used in this study are part of a much larger collection of images that were created using the FaceGen 3.2 modular program (Todorov and Oosterhoof, 2011; Todorov et al., 2013), see Figure 1. The computer-generated facial images were created by varying face shape and reflectiveness and then identifying facial structure features provided by unprompted individuals when presented with computer-generated emotionally neutral faces (Oosterhof and Todorov, 2008; Todorov et al., 2008; Todorov and Oosterhoof, 2011; Todorov and Oh, 2021). The ratings were then gathered and entered into a principal components analysis (PCA) to reduce data dimensionality.

Our study used three facial images from the Todorov threat domain (Todorov, 2018). The three chosen images (high threat, neutral threat, and low threat) have been validated by previous research as relevant to the threat domain and as varying in levels of perceived threat (Todorov et al., 2008, 2013; Todorov and Oosterhoof, 2011). The computer-generated faces were absent of jewelry, obvious emotional facial expression, and head and facial hair to limit confounding factors. Todorov and colleagues used computer-generated Caucasian-looking faces to avoid racial stereotyping, and with a bias toward bald male faces, as they look more natural than bald female faces (Todorov et al., 2008; Verosky and Todorov, 2010). Inter-rater agreement for the threat facial images using a nine-point Likert scale (*1 =* “*not at all threatening*” *to 9 =* “*extremely threatening*”), *N* = 21, *r* = 0.26, reliability = 0.87 (Todorov and Oosterhoof, 2011).

### 2.3. Measures

#### 2.3.1. Social perceptions

For each of the three computerized images, participants were presented with four questions that addressed the following social perceptions; *attractiveness*, *honesty*, *pleasing to look at*, and *threatening*. Using a seven-point Likert scale response format, participants answered the following questions; “How *attractive* is this person?” (*1 = “Extremely Unattractive” to 7 = “Extremely Attractive”*); “How *honest* is this person?” (*1 = “Extremely Dishonest” to 7 = “Extremely Honest”*); “The face in this image is *pleasing to look at*” (*1 = “Strongly Disagree” to 7 = “Strongly Agree”*); “How *non-threatening* or *threatening* is this person?” (1 *= “Extremely Non-Threatening” to 7 = “Extremely Threatening”*).

#### 2.3.2. Emotion label identification

While the faces were designed to be neutral with regard to facial expression, it is possible that participants perceived an expression other than neutral. For this reason, an emotion label for each face was assessed. Participants were instructed to “*As quickly as possible, please choose ONE emotion that best describes the emotion of the individual in the photograph*” (Forced choice options; *angry*, *disgusted*, *fearful*, *happy*, *sad*, *surprised*, and *neutral*).

### 2.4. Procedure

After accessing the study on the MTurk website, participants were directed to a Qualtrics survey. Participants were required to verify their age and consent before beginning the survey. Participants were then directed to answer a series of questions about three images: a high threat, neutral threat, and a low threat facial image from Todorov’s validated threat facial database, as previously mentioned. Participants were asked to make inferences about each of these faces on the social perception characteristics listed above.

The images and questions were presented in a randomized order for each participant. Once the survey was completed, the participants were debriefed, given directions for payment, and thanked for their participation.

## 3. Results

The cross-cultural comparison of the social perception variables was assessed using a 3 (facial images; low, neutral, and high threat) × 2 (culture; India, United States) mixed design (within-subjects variable of facial images, between-subjects variable of culture) ANOVA. A Chi square test and Multinomial Generalized Linear Model with a Bonferroni correction for *post hoc* analyses were used for the emotion label comparisons.

### 3.1. Cross-cultural analyses

#### 3.1.1. Social perceptions

The 3 × 2 mixed design ANOVA revealed a significant interaction of facial image and culture for the social perception of *threat* [*F*(2, 796) = 24.7, *p* < 0.001, *Eta^2^* = 0.022]. The social perceptions of *attractive*, *honesty,* and *pleasing* were non-significant. Table 2 shows the post-hoc results for perceived threat. Indian participants perceived the high threat image to be less threatening than the United States participants, while the Indian and United States differences for the neutral threat and low threat images were non-significant (*p* > 0.05). These results generally support Hypothesis 1; both cultural samples perceived the same pattern of inferences for attractiveness, pleasing-to-look-at and honesty in all three threat conditions, and their threat inferences were the same in two of the three threat conditions. Possible reasons for the difference between the Indian and United States participants in the high threat condition may have been due to the differences between the two groups regarding the emotion labels assigned to the high threat faces. Thirty-three percent of United States participants identified the emotion of the high threat face as angry, whereas 22% of Indian participants identified the high threat face as angry. Additionally, the percentage of Indian participants who identified the high threat face as one of the five remaining emotions (disgust, fear, happy, sad, and surprise) was greater than for the United States participants, indicating a possible reliance on the emotion label as an indicator of threat and not the facial expression.

**Table 2:** *Post hoc* comparisons for the social perception of threat: *Mean (M)* and *Mean Differences (MD)* for Indian and United States sample and condition.

Condition	Indian *M*	United States *M*	*MD*	*SE*	*t*	*p*
Low threat	5.46	5.71	−0.25	0.13	−2.02	0.33
Neutral threat	5.31	5.17	0.15	0.12	1.17	0.85
High threat	4.08	3.20	0.88	0.14	6.30	< 0.001

df = 398.

#### 3.1.2. Emotion inferences

While both Indian and United States participants labeled all three levels of the threat face as neutral more often than not, differences can be seen in the percent of neutral labels as stated above (see Table 3). On average, the United States participants were more likely to rate all three threat faces as neutral than the Indian participants, indicating that the overall perceived neutrality of the three threat images varied depending on culture.

**Table 3:** Cross-cultural comparison of emotion label percentages for high threat, neutral, and low threat faces.

	Low threat		Neutral		High threat	
	India	United States	India	United States	India	United States
Anger	0.5	1.0	0.5	2.5	22.0	32.5
Disgust	3.0	2.0	2.5	2.0	8.0	5.0
Fear	3.0	4.5	3.5	2.0	7.0	1.0
Happy	35.5	21.5	38.5	12.5	14.0	3.0
Sad	9.0	6.0	8.5	2.0	10.5	3.5
Surprised	10	1.5	3.0	1.5	4.0	0.5
Neutral	39	63.5	43.5	77.5	34.5	54.5

In addition to being more likely to label the faces as neutral, similar patterns were observed when participants labeled the emotion as something other than neutral. For the low and neutral threat faces, both Indian and United States participants were more likely to label the face as happy than any other emotion. For the high threat face, both groups of participants were more likely to rate the face as angry. While the emotion labels were similar, cultural differences did exist and are discussed below.

Using a Chi Square analysis, the differences between the Indian and US participants were significant with regard to the perception of emotion in the low threat image [*X^2^*(6, *N* = 400) = 33.7, *p* < 0.01], neutral threat image [*X^2^*(*6*, *N = 400*) *=* 58.3, *p* < 0.01], and the high threat image [*X^2^*(*6*, *N = 400*) *=* 50.1, *p* < 0.01].

As stated previously, the two most common emotions selected by both groups of participants for the low threat face were happy and neutral. A larger percentage of the Indian participants rated the low threat faces as happy (35.5%) than the United States participants (21.5%) while a larger percentage of the United States participants rated the face as neutral (63.5%) than the Indian participants (39%). Additionally, the emotion label of surprise was used by Indians (10%) and to a much lesser extent by United States participants (1.5%). As seen in Table 4, using a Multinomial Generalized Linear Model with a Bonferroni correction, the post-hoc analysis revealed a significant difference between the Indian and United States participants with regard to the emotion labels of happy, neutral, and surprise.

**Table 4:** Cross-cultural comparison of emotion labels for low threat.

Response groups	India/United States	*SE*	*z*	*p*
	Mean difference			
Angry	−0.005	0.009	−0.580	0.573
Disgusted	0.010	0.016	0.641	0.534
Fearful	−0.015	0.019	−0.790	0.445
Happy	0.140	0.045	3.139	< 0.01
Sad	0.030	0.026	1.141	0.276
Surprised	0.085	0.023	3.714	0.003
Neutral	−0.245	0.048	−5.056	< 0.001

Multinomial generalized linear model, *post hoc* analysis, and Bonferroni correction.

As seen in Table 5, a *post hoc* analysis revealed a significant difference between the Indian and United States participants with regard to the emotion labels of happy, neutral, and sad for the neutral threat face. A similar pattern to the low threat face emerged; a larger percentage of Indian participants rated the neutral face as happy (38.5%) than the United States participants (12.5%). A larger percentage of the United States participants rated the face as neutral (77.5%) than the Indian participants (43.5%). Additionally, the emotion label of sad was used by both Indian (8.5%) and United States participants (2%).

**Table 5:** Cross-cultural comparison of emotion labels for neutral threat.

Response groups	India/United States difference	*SE*	*z*	*p*
Angry	−0.020	0.012	−1.651	0.125
Disgust	0.005	0.015	0.337	0.742
Fear	0.015	0.016	0.918	0.377
Happy	0.260	0.042	6.250	< 0.001
Sad	0.065	0.022	2.946	< 0.05
Surprised	0.015	0.015	1.013	0.331
Neutral	−0.340	0.046	−7.418	< 0.001

Multinomial generalized linear model, *post hoc* analysis, and Bonferroni correction.

With regard to the high threat face, the two most common emotions selected by both groups of participants for the high threat face were anger and neutral. A smaller percentage of the Indian participants rated the high threat face as angry (22%) than the United States participants (33.0%). A smaller percentage of the Indian participants rated the high threat faces as neutral (34.5%) than the United States participants (54.5%). Additionally, a higher percentage of the Indian participants labeled the high threat face as fear (Indian participants 7%; United States participants 1%), happy (Indian participants 14%; United States participants 3%), sad (Indian participants 10.5%; United States participants 3.5%) and surprised (Indian participants 4%; United States participants 0.5%), see Table 6 for these differences.

**Table 6:** Cross-cultural comparison of emotion labels for high threat.

Response groups	India/United States difference	*SE*	*z*	*p*
Angry	−0.105	0.044	−2.37	<0.05
Disgust	0.030	0.025	1.22	0.246
Fear	0.060	0.019	3.10	<0.01
Happy	0.110	0.027	4.02	<0.01
Sad	0.070	0.025	2.77	<0.05
Surprised	0.035	0.015	2.38	<0.05
Neutral	−0.200	0.049	−4.11	0.001

Multinomial generalized linear model, *post hoc* analysis, and Bonferroni correction.

## 4. Discussion

Results from this study lead to several conclusions. First, the results expand the usefulness of computer-generated faces as stimuli in facial inferencing studies. These faces can generate significant results from cross-cultural comparisons. Different structural features on these faces generate different emotion and social trait inferences, regardless of culture. Second, the use of presumably neutral computer-generated faces in this study adds to previous findings that neutral facial expressions can be emotionally labeled, and can lead to inferred traits, based on facial structure features as stimuli. The use of facial structure features is further specified by results from a study by Fox and Damjanovic (2006), which indicated that variations in inferred threat can be detected simply by variations in eye features, rather than by the overall face. Third, the threat variations in the computer-generated faces can lead to both evolution-based and culture-based explanations of the facial inferencing results, as indicated in this study’s hypotheses.

The results support Hypothesis 1; the cross-cultural results of this study indicate that facial structural features are used by members of different cultures to make the same inferences in relation to varying levels of threat in neutral faces, with the exception of threat perception in the high-threat face.

The results, for the most part, support the universality hypothesis. Although there was variability in answer choices for the Indian and United States participants, the average answers of both groups for each question were primarily determined by facial structure differences, not by culture. The results appear to demonstrate that humans, regardless of culture, can use basic structural features of the face to make similar inferences even when those faces lack obvious emotional expressions. Apparently, the motivation to make inferences about a person’s internal characteristics is so strong that people will use whatever features are available as a basis for inferences. If emotional expressions and social category identifiers (such as age and gender) are absent or ambiguous, inferences will be made from structural features.

The results also mainly support Hypothesis 2. United States participants more accurately labeled the European looking neutral expression faces as neutral emotionally than did the Indian participants. These results support the In-group advantage concept. However, cultural differences in the results were not just due to the In-group advantage. Indian participants made more positive emotional and social trait inferences to all three images than the United States participants. According to the cross-cultural results, Indian participants descriptively perceived all three images as happier, more attractive, more pleasing to look at, more honest and, in particular, significantly less threatening in the high-threat image.

Perhaps the explanation for these findings can be found by examining India-United States differences in the Collectivism/Individualism Scale (Hofstede, 2019a). The India and United States overall country scores on this dimension vary considerably (Hofstede, 2019b). On the scale (0–100), the United States had the highest country score on Individualism (91), while India was much more Collectivist (48). Individuals in collectivist cultures have more interdependent self-construals and therefore have a greater sense of positive connectedness to others (Markus and Kitiyama, 1991; Kapoor et al., 2003; Keller and Otto, 2009). There is a greater need for cooperation in collectivist cultures to preserve harmonious relations in groups and therefore to express other-focused, socially engaging emotions (Verma and Triandis, 1999). Children are made aware of this need, and as they grew up they learn display rules about expressing positive emotions and suppressing negative emotions in social interactions (Joshi and MacLean, 1994; Raval et al., 2007; Wilson et al., 2011). Presumably, this translates into expecting others to do the same, leading the Indian participants to perceive all of the faces more positively.

There are two possible alternative explanations for the Indians’ more positive perceptions of European neutral faces. India was ruled by the British for over 200 years, and it is possible that the positive evaluation of European facial features is due to Indian internalization of European superiority. This explanation is counteracted by the fact that British rule of India ended in 1948, 75 years ago. Presumably, 96.5% of all our Indian participants were born after 1948 (see Table 1), when India was ruled by Indians, and therefore much less likely to be influenced by any perceived European superiority.

The second alternative explanation indicates that the collectivism–individualism dimension by itself may not be sufficient to explain the Indian positivity findings. Sinha and Tripathi (1994) state that Indian culture widely incorporates both collectivist and individualistic elements. India, however, is not just a collectivist culture; it is a vertical collectivist culture, meaning that it is high in what is called “power distance.” Power distance refers to both the reality and acceptance of a high level of social inequity in a society. Applying Hofstede’s power distance dimension to compare United States with India, the United States measures 40, low power distance, compared to India’s 77, a much higher power distance (Hofstede, 2019a). Indians may both enact and perceive more positive facial expressions because of status concerns.

### 4.1. Limitations and future directions

In general, terms facial inferencing is only part of the overall domain of social perception. How humans perceive each other is based on what is said, non-verbal behavior outside of the face, facial expressions and structural features, eye behavior, and environmental cues surrounding social interactions. Past studies (Mehrabian and Ferris, 1967; Mehrabian and Weiner, 1967) have shown that attempts to understand the intentions, emotions, and traits of another person are most heavily influenced by non-verbal behavior, and within non-verbal behavior by facial expressions and features, including eye behavior. Given this, a continuing research focus on facial inferencing as an important part of social perception is warranted, while not ignoring the contributions of other avenues of social perception.

We identified three specific potential limitations in our research. First, as is always the case with online survey research, lack of control over environmental distraction and participant focus may be responsible for response variability. Possible ways to check for distraction and focus include the addition of attention questions throughout the survey. These questions are designed to indicate to researchers how engaged participants are by presenting random instructions that appear to go against obvious answers. Our online survey did not include attention questions and as a result, we were not able to check for distraction and focus. However, due to the patterns within and between our two fairly large cultural samples, we are reasonably confident distraction was not a primary confound.

English was presumably a second language for most of the Indian participants (only 10.67% of the 2011 Indian census were identified as speakers of English; Ministry of Home Affairs, Government of India, 2011; Census), which may have led to some confusion about the wording in the questions and responses. Additionally, a complete demographic analysis of the Indian and United States samples was not possible. For example, it may have been the case that the Indian sample contained US participants and *vice-versa*, even though the MTurk advertisement specifically stated that participants be either “American” and living in the United States or “Indian” and living in India. Future research should focus on a more diverse cultural sample in order to gain a more accurate understanding of cultural similarities and differences. A third limitation was the lack of a specific individual-oriented measurement of the Collectivism/Individualism Scale.

Finally, as a future research direction, even though facial structure features are now being rigorously studied, it is still not completely clear which facial structure feature is focused on most when people make various trait inferences. A comparative focus on the specific facial structure features in studies such as this may provide more insight into the similarities and differences in the inference process across cultures.

### 4.2. Summary

The findings of this study reflect an interaction between evolution and culture. Both cultural samples accurately distinguished the varying threat levels demonstrated in the three faces. The participants from both cultures made evolutionarily appropriate inferences to each face, inferring more negativity in the high-threat face and more positivity in the low-threat face. Our results align with the general trend of cross-cultural results on facial inferencing. The current consensus appears to be that facial inferencing is not either/or. Facial inferencing cannot be completely explained by universal, evolutionary-based processes, nor can it be completely explained by cultural differences (Fernandez-Dols and Russell, 2017). Evolution and culture interact as causes of facial inferencing, and it should be the goal of future research to continue to assess the contributions of both.

## Data availability statement

The datasets presented in this article are not readily available because permission from the authors’ IRB is required. Requests to access the datasets should be directed to AS at stahelsa@cwu.edu.

## Ethics statement

The studies involving human participants were reviewed and approved by the Central Washington University, Human Subjects Review Council, https://www.cwu.edu/hsrc/. Authors are faculty at Central Washington University. Written informed consent for participation was not required for this study in accordance with the national legislation and the institutional requirements.

## Author contributions

All authors listed have made a substantial, direct, and intellectual contribution to the work and approved it for publication.

## Conflict of interest

The authors declare that the research was conducted in the absence of any commercial or financial relationships that could be construed as a potential conflict of interest.

## Publisher’s note

All claims expressed in this article are solely those of the authors and do not necessarily represent those of their affiliated organizations, or those of the publisher, the editors and the reviewers. Any product that may be evaluated in this article, or claim that may be made by its manufacturer, is not guaranteed or endorsed by the publisher.
